# Supplementary figures and images for: First identification of genotypes of Enterocytozoon bieneusi (Microsporidia) among symptomatic and asymptomatic children in Mozambique
Source: PLoS Negl Trop Dis. 2020 Jun 30;14(6):e0008419. doi: 10.1371/journal.pntd.0008419 (PMC7357779; doi:10.1371/journal.pntd.0008419)

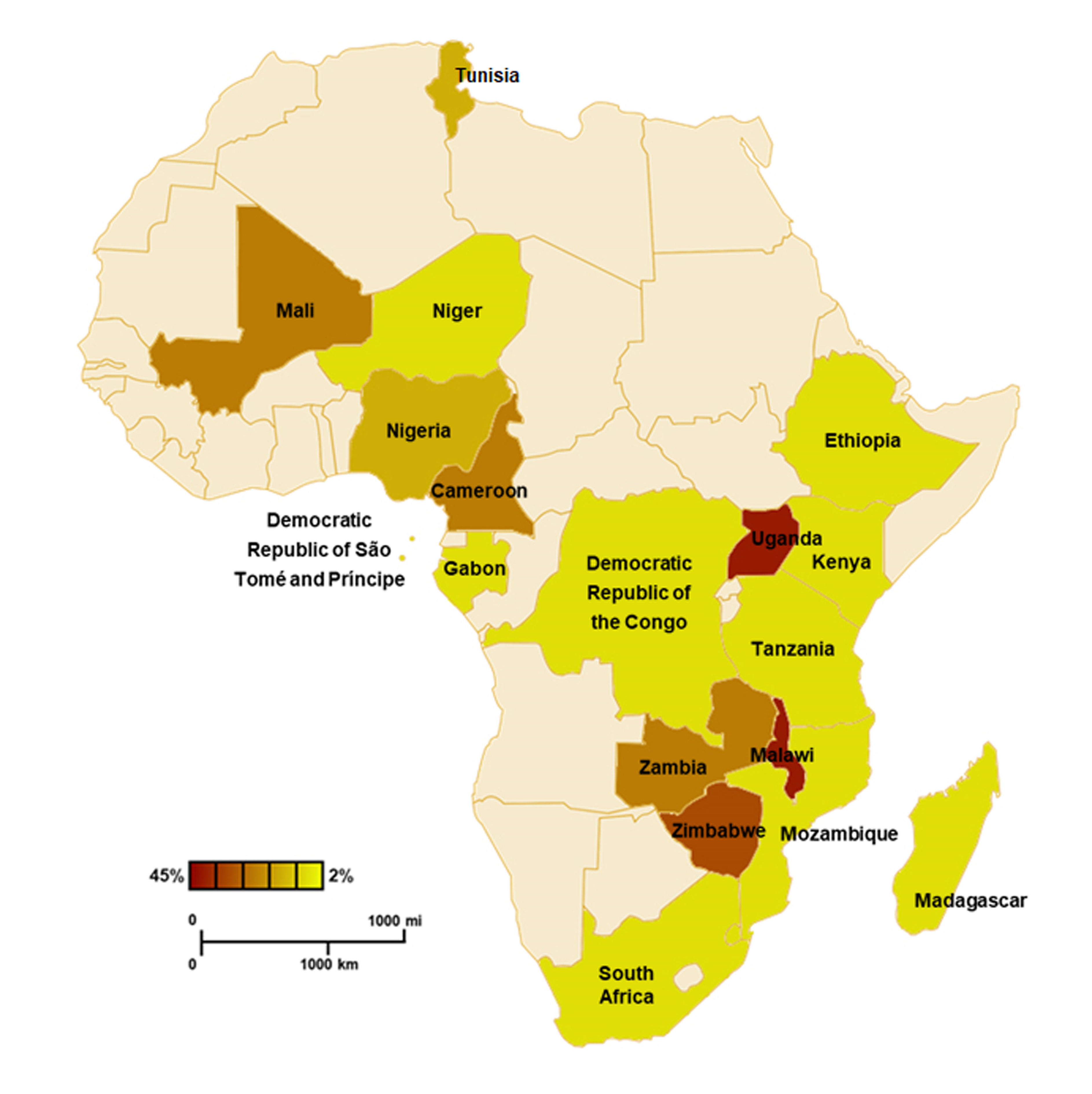

Supplement: S1 Fig — Average values for symptomatic and asymptomatic individuals are represented according to reported infection rates summarized in Table 1. (TIF) [file pntd.0008419.s001.tif]
