# Supplementary material for: First identification of genotypes of Enterocytozoon bieneusi (Microsporidia) among symptomatic and asymptomatic children in Mozambique
Source: PLoS Negl Trop Dis. 2020 Jun 30;14(6):e0008419. doi: 10.1371/journal.pntd.0008419 (PMC7357779; doi:10.1371/journal.pntd.0008419)
Supplement: S1 Table — (DOCX) [file pntd.0008419.s002.docx]

**S1 Table. Main socio-demographic features and risk factors of the asymptomatic schoolchildren population (*n* = 807) investigated in Zambézia province (Mozambique), 2017‒2019.**

|  |  |  |  | **Gender** | | **Age group (years)** | | | **Contact with livestock and/or poultry** | | **Contact with companion animals** | | **Main source of drinking water** | | | **Defecation place** | |
| --- | --- | --- | --- | --- | --- | --- | --- | --- | --- | --- | --- | --- | --- | --- | --- | --- | --- |
| **District** | **School** | **Area** | **Total** | **Male** | **Female** | **0‒5** | **6‒10** | **11‒14** | **Yes** | **No** | **Yes** | **No** | **River** | **Tap** | **Well** | **Latrine** | **Outside** |
| Alto Molócue | 1 | Rural | 44 | 27 | 17 | 4 | 40 | 0 | 0 | 44 | 3 | 41 | 0 | 8 | 36 | 40 | 4 |
|  | 2 | Rural | 50 | 27 | 23 | 5 | 43 | 2 | 0 | 50 | 3 | 47 | 0 | 4 | 46 | 45 | 5 |
| Gurúe | 3 | Urban | 22 | 13 | 9 | 3 | 14 | 5 | 0 | 22 | 9 | 13 | 5 | 4 | 13 | 20 | 2 |
| Ile | 4 | Rural | 22 | 10 | 12 | 22 | 0 | 0 | 3 | 19 | 3 | 19 | 0 | 0 | 22 | 0 | 22 |
|  | 5 | Rural | 24 | 13 | 11 | 6 | 16 | 2 | 1 | 23 | 4 | 20 | 0 | 0 | 24 | 18 | 6 |
| Lugela | 6 | Rural | 31 | 13 | 18 | 0 | 20 | 11 | 2 | 29 | 7 | 24 | 4 | 0 | 27 | 31 | 0 |
|  | 7 | Rural | 88 | 56 | 32 | 8 | 46 | 34 | 4 | 84 | 14 | 74 | 8 | 0 | 80 | 80 | 8 |
| Mocuba | 8 | Rural | 60 | 27 | 33 | 1 | 50 | 9 | 0 | 60 | 8 | 52 | 0 | 0 | 60 | 59 | 1 |
|  | 9 | Urban | 47 | 19 | 28 | 7 | 38 | 2 | 1 | 46 | 12 | 35 | 11 | 4 | 32 | 47 | 0 |
|  | 10 | Rural | 49 | 20 | 29 | 0 | 22 | 27 | 0 | 49 | 9 | 40 | 0 | 0 | 45^a^ | 49 | 0 |
|  | 17 | Rural | 75 | 38 | 37 | 27 | 48 | 0 | 14 | 61 | 9 | 66 | 0 | 0 | 75 | 48 | 27 |
| Mopeia | 11 | Rural | 47 | 26 | 21 | 0 | 37 | 10 | 0 | 47 | 10 | 37 | 3 | 4 | 40 | 47 | 0 |
|  | 12 | Rural | 50 | 28 | 22 | 0 | 23 | 27 | 0 | 50 | 10 | 40 | 3 | 3 | 44 | 50 | 0 |
| Morrumbala | 13 | Rural | 50 | 23 | 27 | 0 | 42 | 8 | 1 | 49 | 5 | 45 | 1 | 1 | 48 | 50 | 0 |
|  | 18 | Rural | 48 | 16 | 32 | 15 | 25 | 8 | 11 | 37 | 23 | 25 | 0 | 0 | 48 | 33 | 15 |
| Namacurra | 14 | Rural | 30 | 13 | 17 | 5 | 25 | 0 | 0 | 30 | 8 | 22 | 0 | 16 | 14 | 26 | 4 |
| Nicoadala | 15 | Rural | 30 | 15 | 15 | 5 | 23 | 2 | 0 | 30 | 7 | 23 | 0 | 23 | 7 | 27 | 3 |
| Quelimane | 16 | Urban | 40 | 17 | 23 | 0 | 7 | 33 | 0 | 40 | 8 | 32 | 0 | 40 | 0 | 40 | 0 |
| **Total** |  |  | 807 | 401 | 406 | 108 | 519 | 180 | 37 | 770 | 152 | 655 | 35 | 187 | 581^a^ | 710 | 97 |

^a^ Four results missing.
