# Supplementary material for: First identification of genotypes of Enterocytozoon bieneusi (Microsporidia) among symptomatic and asymptomatic children in Mozambique
Source: PLoS Negl Trop Dis. 2020 Jun 30;14(6):e0008419. doi: 10.1371/journal.pntd.0008419 (PMC7357779; doi:10.1371/journal.pntd.0008419)
Supplement: S2 Table — (DOCX) [file pntd.0008419.s003.docx]

**S2 Table. Main socio-demographic features and risk factors of the symptomatic schoolchildren population (*n* = 290) attended at public health centres in Zambézia province (Mozambique), 2017‒2018.**

|  |  |  |  | **Gender** | | **Age group (years)** | | | **Contact with livestock and/or poultry** | | **Contact with companion animals** | | **Main source of drinking water** | | | **Defecation place** | |
| --- | --- | --- | --- | --- | --- | --- | --- | --- | --- | --- | --- | --- | --- | --- | --- | --- | --- |
| **District** | **Healthcare Centre** | **Area** | **Total** | **Male** | **Female** | **0-5** | **6-10** | **11-14** | **Yes** | **No** | **Yes** | **No** | **River** | **Tap** | **Well** | **Latrine** | **Outside** |
| Alto Molócue | Sede | Rural | 50 | 33 | 17 | 15 | 34 | 1 | 13 | 37 | 13 | 37 | 4 | 11 | 35 | 35 | 15 |
| Gurúe | Sede | Urban | 28 | 18 | 10 | 16 | 9 | 3 | 2 | 26 | 7 | 21 | 0 | 5 | 23 | 12 | 16 |
| Ile | Sede | Rural | 15 | 10 | 5 | 4 | 10 | 1 | 1 | 14 | 4 | 11 | 0 | 0 | 15 | 11 | 4 |
| Lugela | Sede | Rural | 52 | 22 | 30 | 20 | 22 | 10 | 18 | 34 | 18 | 34 | 7 | 0 | 45 | 32 | 20 |
| Mocuba | Sede | Urban | 43 | 18 | 25 | 13 | 16 | 14 | 0 | 43 | 4 | 39 | 1 | 39 | 3 | 37 | 6 |
| Quelimane | 17 de Setembro | Urban | 29 | 13 | 16 | 8 | 15 | 6 | 1 | 28 | 12 | 17 | 0 | 29 | 0 | 27 | 2 |
|  | Hospital Central | Urban | 73 | 32 | 41 | 22 | 37 | 14 | 6 | 67 | 19 | 54 | 0 | 73 | 0 | 69 | 4 |
| **Total** |  |  | 290 | 146 | 144 | 98 | 143 | 49 | 41 | 249 | 77 | 213 | 12 | 157 | 121 | 223 | 67 |
