# Supplementary material for: First identification of genotypes of Enterocytozoon bieneusi (Microsporidia) among symptomatic and asymptomatic children in Mozambique
Source: PLoS Negl Trop Dis. 2020 Jun 30;14(6):e0008419. doi: 10.1371/journal.pntd.0008419 (PMC7357779; doi:10.1371/journal.pntd.0008419)
Supplement: S3 Table — (DOCX) [file pntd.0008419.s004.docx]

**S3 Table. Main socio-demographic features and risk factors of the asymptomatic children population (*n* = 109) investigated in Maputo province (Mozambique), 2016‒2018.**

|  |  |  |  | **Gender** | | **Age group (months)** | | | **Contact with livestock and/or poultry** | | **Contact with companion animals** | | **Main source of drinking water** | | | **Defecation place** | |
| --- | --- | --- | --- | --- | --- | --- | --- | --- | --- | --- | --- | --- | --- | --- | --- | --- | --- |
| **District** | **Hospital** | **Area** | **Total** | **Male** | **Female** | **0‒11** | **12‒23** | **24‒59** | **Yes** | **No** | **Yes** | **No** | **River** | **Tap^$^** | **Well^*^** | **Latrine** | **Outside** |
| Manhiça | MDH | Rural | 70 | 39 | 31 | 15 | 24 | 30^a^ | 24^b^ | 19^b^ | 7^b^ | 36^b^ | 1 ^b^ | 34^b^ | 8^b^ | 66 ^e^ | 0 ^e^ |
|  | XRH | Rural | 39 | 20 | 19 | 19 | 18 | 11 | 20^c^ | 13^c^ | 11^c^ | 22^c^ | 0^c^ | 31^c^ | 2^c^ | 38 ^f^ | 0 ^f^ |
| **Total** |  |  | 109 | 59 | 50 | 25 | 42 | 41^a^ | 44^d^ | 32^d^ | 18^d^ | 58^d^ | 1^d^ | 65 ^d^ | 10 ^d^ | 104 ^g^ | 0 ^g^ |

MDH: Manhiça District Hospital; XRH: Xinavane Rural Hospital.

**^$^** Tap: public, at home or from a bore hole.

^*^ Well: covered (protected) or not (unprotected).

^a^ One result missing.

^b^ Twenty-seven results missing.

^c^ Six results missing.

^d^ Thirty-three results missing.

^e^ Two results missing

^f^ Three results missing

^g^ Five results missing
