# Supplementary material for: First identification of genotypes of Enterocytozoon bieneusi (Microsporidia) among symptomatic and asymptomatic children in Mozambique
Source: PLoS Negl Trop Dis. 2020 Jun 30;14(6):e0008419. doi: 10.1371/journal.pntd.0008419 (PMC7357779; doi:10.1371/journal.pntd.0008419)
Supplement: S4 Table — (DOCX) [file pntd.0008419.s005.docx]

**S4 Table. Main socio-demographic features and risk factors of the symptomatic children population (*n* = 41) investigated in Maputo province (Mozambique), 2016–2018.**

|  |  |  |  | **Gender** | | **Age group (months)** | | | **Contact with livestock and/or poultry** | | **Contact with companion animals** | | **Main source of drinking water** | | | **Defecation place** | |
| --- | --- | --- | --- | --- | --- | --- | --- | --- | --- | --- | --- | --- | --- | --- | --- | --- | --- |
| **District** | **Hospital** | **Area** | **Total** | **Male** | **Female** | **0‒11** | **12‒23** | **24‒59** | **Yes** | **No** | **Yes** | **No** | **River** | **Tap^$^** | **Well**^*^ | **Latrine** | **Outside** |
| Manhiça | MDH | Rural | 24 | 13 | 11 | 9 | 7 | 8 | 13 | 11 | 1 | 23 | 0 | 22 | 2 | 23 ^a^ | 0 ^a^ |
|  | XRH | Rural | 17 | 7 | 10 | 1 | 12 | 4 | 4 | 12^a^ | 2 | 14^a^ | 1 | 11 | 5 | 17 | 0 |
| **Total** | NA | Rural | 41 | 20 | 21 | 10 | 19 | 12 | 17^b^ | 23 | 3^b^ | 37 | 1 | 33 | 7 | 40 ^a^ | 0 ^a^ |

NA: Not applicable; MDH: Manhiça District Hospital; XRH: Xinavane Rural Hospital.

**^$^** Tap: public, at home or from a bore hole

^*^ Well: covered (protected) or not (unprotected)

^a^ One result missing.

^b^ One result did not specify the species of animal.
