# Supplementary material for: First identification of genotypes of Enterocytozoon bieneusi (Microsporidia) among symptomatic and asymptomatic children in Mozambique
Source: PLoS Negl Trop Dis. 2020 Jun 30;14(6):e0008419. doi: 10.1371/journal.pntd.0008419 (PMC7357779; doi:10.1371/journal.pntd.0008419)
Supplement: S5 Table — Highlighted in bold are African countries. (DOCX) [file pntd.0008419.s006.docx]

**Table S5. Summary of all *Enterocytozoon bieneusi* genotypes reported in Africa including host and geographic range.** Highlighted in bold are African countries.

| **Genotype (synonym)** | **Host(s)^a^** | **Geographic distribution** | **Reference** |
| --- | --- | --- | --- |
| B (Type I) | Human | Australia, **Cameroon**, England, France, Germany, Netherlands, **Nigeria**, Switzerland, **Tunisia** | [1-9] |
| CAF2 | Human | **Gabon, Nigeria** | [1,10] |
| CAF3 | Human | **Gabon** | [1] |
| CAF4 | Human | **Cameroon, Gabon** | [1] |
| KIN3 | Human | **Congo** | [11] |
| NIA1 | Human | Brazil, **Congo**, **Niger** | [11-14] |
| Nig1 | Human | **Nigeria** | [10] |
| Nig2 | Human | **Nigeria** | [10] |
| Nig3 | Human | **Nigeria** | [10] |
| Nig4 | Human | **Nigeria** | [10] |
| Nig5 | Human | **Nigeria** | [10] |
| S1 | Human | **Malawi** | [3] |
| S2 | Human | **Malawi, Mozambique** | [3]; This study |
| S3 | Human | **Malawi** | [3] |
| S4 | Human | **Malawi** | [3] |
| UG2145 | Human | **Malawi**, **Uganda** | [3,15] |
| HhMzEb1 | Human | **Mozambique** | This study |
| A (Peru1, KIN-2) | Human | **Cameroon**, **Congo**, **Democratic Republic of São Tomé and Príncipe**, **Gabon**, Germany, India, Netherlands, **Niger**, **Nigeria**, Peru, Portugal, Switzerland, Slovak Republic, Thailand | [1,3,7,10-12,16-26] |
|  | NHP | **Kenya** | [27] |
|  | Dog | Spain | [28] |
|  | Bird | Czech Republic | [29] |
| Peru 11 (Peru12) | Human | China, **Mozambique**, Peru, Thailand | [16-18,30-32]; This study |
|  | NHP | China, **Kenya** | [27,33-35] |
|  | Cat | Spain | [36] |
|  | Raccoon | USA | [37] |
|  | Mouse | USA | [37] |
|  | Rabbit | USA | [37] |
|  | Bird | Brazil | [38] |
| Peru7 | Human | Peru | [16-18] |
|  | NHP | **Kenya** | [27] |
| CAF1 (KIN-1, PEbE) | Human | **Cameroon**, **Congo**, **Democratic Republic of São Tomé and Príncipe**, **Gabon**, **Niger** | [1,11,12,23,39] |
|  | Cattle | **Ethiopia** | Unpublished^b^ |
|  | Goat | China | [40] |
|  | Pig | **Gabon**, Korea | [1,41] |
|  | Deer | China | Unpublished^c^ |
| Type IV (K, Peru2, BEB5, CMITS1, BEB-var, PtEB III) | Human | **Cameroon**, China, **Democratic Republic of São Tomé and Príncipe**, England, France  **Gabon**, Iran, **Malawi**, **Mozambique**, Netherlands, **Niger**, **Nigeria**, Peru, Portugal, **Uganda** | [1,4-5,8,10,12,15-20,23,30,39,42-45]; This study |
|  | NHP | China | [34,46] |
|  | Cattle | Korea, Portugal, USA | [47-49] |
|  | Cat | China, Colombia, Germany, Japan  Portugal, Turkey | [50-57] |
|  | Dog | China, Colombia | [58,59] |
|  | Bear | USA | [37] |
|  | Chipmunk | USA | [37] |
|  | Groundhog | USA | [37] |
|  | Mouse | USA | [37] |
|  | Squirrel | USA | [37] |
|  | Birds | Brazil, Spain | [28,38] |
|  | Snake | China | [60] |
| D (PigEBITS9, WL8, Peru9, CEbC, PTEb VI) | Human | Brazil, **Cameroon**, China, **Congo**, **Democratic Republic of São Tomé and Príncipe**, England, **Gabon**, India, Iran, **Malawi**, Netherlands, **Niger**, **Nigeria** Peru, Poland, Portugal, Russia, Spain, Thailand, **Tunisia**, Vietnam | [1-3,8,10-13,16-21,23,30-32,43,44,61-66] |
|  | NHP | China, **Kenya**, USA | [27,33,46,67-69] |
|  | Cattle | Argentina, Brazil, China, Korea, **South Africa** | [49,70-75] |
|  | Sheep | China | [103] |
|  | Goat | China | [103] |
|  | Pig | China, Czech Republic, Japan, Thailand, USA | [63,76-80] |
|  | Horse | **Algeria**, China, Colombia, Czech Republic | [81-84] |
|  | Cat | Brazil, China, Spain, Thailand, Turkey | [36,52-54,56,58,85,86] |
|  | Dog | China, Poland, Portugal | [51,54,58,87] |
|  | Wild boar | Czech Republic, Slovak Republic | [88] |
|  | Takin | China | [89] |
|  | Beaver | USA | [90] |
|  | Fox | China, Spain, USA | [28,90-92] |
|  | Muskrat | USA | [90] |
|  | Raccoon | China, USA | [54, 67,90-92] |
|  | Otter | USA | [37] |
|  | Rabbit | Spain | [28] |
|  | African lion | China | [67] |
|  | Asian golden cat | China | [67] |
|  | Mouse | Czech Republic, Germany | [93] |
|  | Bird | Abu Dhabi, Brazil, Iran, | [38,94,95] |
| S6 | Human | **Malawi** | [3] |
|  | Mice | Czech Republic, Germany | [93] |
| Peru8 | Human | China, **Malawi**, **Nigeria**, Peru, **Tunisia** | [2,3,10,16-18,30] |
|  | NHP | China | [33,46] |
|  | Horse | China | [83] |
|  | Dog | China | [58] |
|  | Mouse | Czech Republic, Germany | [93] |
| CZ3 | Human | Czech Republic | [96] |
|  | Horse | **Algeria** | [82] |
|  | Mouse | Czech Republic, Germany | [93] |
| BEB4 (CHN1) | Human | China | [96,97] |
|  | NHP | China | [46] |
|  | Cattle | Argentina, Brazil, China, **South Africa**, USA | [47,48,70-74,97-102] |
|  | Yak | China | [101] |
|  | Pig | China | [97] |
| EbpA (F) | Human | China, Czech Republic, **Nigeria** | [10,31,96] |
|  | NHP | China | [46] |
|  | Cattle | Brazil, China, Germany | [50,71,72,74] |
|  | Goat | China | [40,103] |
|  | Pig | Brazil China, Czech Republic  Germany, Japan, Switzerland, Thailand, USA | [50,63,76-80,104-109] |
|  | Horse | China, Czech Republic | [81,83] |
|  | Dog | China | [58] |
|  | Mouse | Czech Republic, Germany | [93] |
|  | Wild boar | Czech Republic; Poland | [88] |
|  | Bird | Brazil, Czech Republic | [29,110] |
| I (BEB2, CEbE) | Human | China | [97] |
|  | NHP | China | [33] |
|  | Cattle | Argentina, Brazil, China, Czech Republic, Germany, Korea, **South Africa**, USA | [47-50,70-75,97-102,104,111,112] |
|  | Pig | Spain | [28] |
|  | Cat | China | [58] |
|  | Yak | China | [101] |
|  | Deer | USA | [113] |
| CHN4 | Human | China, **Uganda** | [97,114] |
|  | Cattle | China | [97] |
| WL7 | Human | **Nigeria** | [20] |
|  | Beaver | USA | [90] |
| S5 | Human | **Malawi** | [3] |
|  | European badger | Spain | [115] |
| KB-1 | NHP | **Kenya** | [27] |
| KB-2 | NHP | **Kenya** | [27] |
| KB-3 | NHP | **Kenya** | [27] |
| KB-4 | NHP | **Kenya** | [27] |
| KB-5 | NHP | **Kenya** | [27] |
| KB-6 | NHP | Kenya | [27] |
| Macaque1 | NHP | China | [34] |
|  | Camel | **Algeria** | [116] |
| Horse1 | Horse | **Algeria**, China, Colombia, Czech Republic | [81-84] |
| Horse2 | Horse | **Algeria**, China, Colombia, Czech Republic | [81-84] |

^a^ NHP: Non-human primate.

^b^ GenBank nucleotide sequence available (KT922239).

^c^ GenBank nucleotide sequence available (KR815514).

**References**

1. Breton J, Bart-Delabesse E, Biligui S, Carbone A, Seiller X, Okome-Nkoumou M, et al. New highly divergent rRNA sequence among biodiverse genotypes of *Enterocytozoon bieneusi* strains isolated from humans in Gabon and Cameroon. J Clin Microbiol. 2007;45:2580-9.
2. Chabchoub N, Abdelmalek R, Breton J, Kanoun F, Thellier M, Bouratbine A, Aoun K. Genotype identification of *Enterocytozoon bieneusi* isolates from stool samples of HIV-infected Tunisian patients. Parasite. 2012;19:147-51.
3. ten Hove RJ, Van Lieshout L, Beadsworth MB, Perez MA, Spee K, Claas EC, et al. Characterization of genotypes of *Enterocytozoon bieneusi* in immunosuppressed and immunocompetent patient groups. J Eukaryot Microbiol. 2009;56:388-93.
4. Liguory O, David F, Sarfati C, Derouin F, Molina JM. Determination of types of *Enterocytozoon bieneusi* strains isolated from patients with intestinal microsporidiosis. J Clin Microbiol. 1998;36:1882-5.
5. Liguory O, Sarfati C, Derouin F, Molina JM. Evidence of different *Enterocytozoon bieneusi* genotypes in patients with and without human immunodeficiency virus infection. J Clin Microbiol. 2001;39:2672-4.
6. Ojuromi OT, Izquierdo F, Fenoy S, Fagbenro-Beyioku A, Oyibo W, Akanmu A, et al. Identification and characterization of microsporidia from fecal samples of HIV-positive patients from Lagos, Nigeria. PLoS One. 2012;7:e35239.
7. Rinder H, Katzwinkel-Wladarsch S, Löscher T. Evidence for the existence of genetically distinct strains of *Enterocytozoon bieneusi*. Parasitol Res. 1997;83:670-2.
8. Sadler F, Peake N, Borrow R, Rowl PL, Wilkins EG, Curry A. Genotyping of *Enterocytozoon bieneusi* in AIDS patients from the north west of England. J Infect. 2002;44:39-42.
9. Stark D, van Hal S, Barratt J, Ellis J, Marriott D, Harkness J. Limited genetic diversity among genotypes of *Enterocytozoon bieneusi* strains isolated from HIV-infected patients from Sydney, Australia. J Med Microbiol. 2009;58:355-7.
10. Akinbo FO, Okaka CE, Omoregie R, Dearen T, Leon ET, Xiao L. Molecular epidemiologic characterization of *Enterocytozoon bieneusi* in HIV-infected persons in Benin City, Nigeria. Am J Trop Med Hyg. 2012;86:441-5.
11. Wumba R, Longo-Mbenza B, Menotti J, Mandina M, Kintoki F, Situakibanza NH, Kakicha MK, Zanga J, Mbanzulu-Makola K, Nseka T, Mukendi JP, Kendjo E, Sala J, Thellier M. Epidemiology, clinical, immune, and molecular profiles of microsporidiosis and cryptosporidiosis among HIV/AIDS patients. Int J Gen Med. 2012;5:603-11.
12. Espern A, Morio F, Miegeville M, Illa H, Abdoulaye M, Meyssonnier V, et al. Molecular study of microsporidiosis due to *Enterocytozoon bieneusi* and *Encephalitozoon intestinalis* among human immunodeficiency virus-infected patients from two geographical areas: Niamey, Niger, and Hanoi, Vietnam. J Clin Microbiol. 2007;45:2999-3002.
13. Feng Y, Li N, Dearen T, Lobo ML, Matos O, Cama V, et al. Development of a multilocus sequence typing tool for high-resolution genotyping of *Enterocytozoon bieneusi*. Appl Environ Microbiol. 2011;77:4822-8.
14. Wumba R, Longo-Mbenza B, Mandina M, Odio WT, Biligui S, Sala J, et al. Intestinal parasites infections in hospitalized AIDS patients in Kinshasa, Democratic Republic of Congo. Parasite. 2010;17:321-8.
15. Tumwine JK, Kekitiinwa A, Nabukeera N, Akiyoshi DE, Buckholt MA, Tzipori S. *Enterocytozoon bieneusi* among children with diarrhea attending Mulago Hospital in Uganda. Am J Trop Med Hyg. 2002;67:299-303.
16. Sulaiman IM, Bern C, Gilman R, Cama V, Kawai V, Vargas D, et al. A molecular biologic study of *Enterocytozoon bieneusi* in HIV-infected patients in Lima, Peru. J Eukaryot Microbiol. 2003;50 Suppl:591-6.
17. Bern C, Kawai V, Vargas D, Rabke-Verani J, Williamson J, Chavez-Valdez R, et al. The epidemiology of intestinal microsporidiosis in patients with HIV/AIDS in Lima, Peru. J Infect Dis. 2005;191:1658-64.
18. Cama VA, Pearson J, Cabrera L, Pacheco L, Gilman R, Meyer S, et al. Transmission of *Enterocytozoon bieneusi* between a child and guinea pigs. J Clin Microbiol. 2007;45:2708-10.
19. Lobo ML, Xiao L, Antunes F, Matos O. Microsporidia as emerging pathogens and the implication for public health: a 10-year study on HIV-positive and -negative patients. Int J Parasitol. 2012;42:197-205.
20. Maikai BV, Umoh JU, Lawal IA, Kudi AC, Ejembi CL, Xiao L. Molecular characterizations of *Cryptosporidium*, *Giardia*, and *Enterocytozoon* in humans in Kaduna State, Nigeria. Exp Parasitol. 2012;131:452-6.
21. Li W, Cama V, Akinbo FO, Ganguly S, Kiulia NM, Zhang X, et al. Multilocus sequence typing of *Enterocytozoon bieneusi*: Lack of geographic segregation and existence of genetically isolated sub-populations. Infect Genet Evol. 2013;14:111-9.
22. Halánová M, Valenčáková A, Malčeková B, Kváč M, Sak B, Květoňová D, Bálent P, Čisláková L. Occurrence of microsporidia as emerging pathogens in Slovak Roma children and their impact on public health. Ann Agric Environ Med. 2013;20:695-8.
23. Lobo ML, Augusto J, Antunes F, Ceita J, Xiao L, Codices V, et al. *Cryptosporidium* spp., *Giardia duodenalis*, *Enterocytozoon bieneusi* and other intestinal parasites in young children in Lobata province, Democratic Republic of São Tomé and Principe. PLoS One. 2014;9:e97708.
24. Leelayoova S, Subrungruang I, Rangsin R, Chavalitshewinkoon-Petmitr P, Worapong J, Naaglor T, et al. Transmission of *Enterocytozoon bieneusi* genotype a in a Thai orphanage. Am J Trop Med Hyg. 2005;73:104-7.
25. Leelayoova S, Piyaraj P, Subrungruang I, Pagornrat W, Naaglor T, Phumklan S, et al. Genotypic characterization of *Enterocytozoon bieneusi*  in specimens from pigs and humans in a pig farm community in Central Thailand. J Clin Microbiol. 2009;47:1572-4.
26. Pagornrat W, Leelayoova S, Rangsin R, Tan-Ariya P, Naaglor T, Mungthin M. Carriage rate of *Enterocytozoon bieneusi* in an orphanage in Bangkok, Thailand. J Clin Microbiol. 2009;47:3739–3741.
27. Li W, Kiulia NM, Mwenda JM, Nyachieo A, Taylor MB, Zhang X, et al. *Cyclospora papionis*, *Cryptosporidium hominis*, and human-pathogenic *Enterocytozoon bieneusi* in captive baboons in Kenya. J Clin Microbiol. 2011;49:4326-9.
28. Galván-Díaz AL, Magnet A, Fenoy S, Henriques-Gil N, Haro M, Gordo FP, et al. Microsporidia detection and genotyping study of human pathogenic *E. bieneusi* in animals from Spain. PLoS One. 2014;9:e92289.
29. Kasicková D, Sak B, Kvác M, Ditrich O. Sources of potentially infectious human microsporidia: molecular characterisation of microsporidia isolates from exotic birds in the Czech Republic, prevalence study and importance of birds in epidemiology of the human microsporidial infections. Vet Parasitol. 2009;165:125-30.
30. Wang L, Zhang H, Zhao X, Zhang L, Zhang G, Guo M, et al. Zoonotic *Cryptosporidium* species and *Enterocytozoon bieneusi* genotypes in HIV-positive patients on antiretroviral therapy. J Clin Microbiol. 2013 Feb;51(2):557-63.
31. Wang L, Xiao L, Duan L, Ye J, Guo Y, Guo M, et al. Concurrent infections of *Giardia duodenalis*, *Enterocytozoon bieneusi*, and *Clostridium difficile* in children during a cryptosporidiosis outbreak in a pediatric hospital in China. PLoS Negl Trop Dis. 2013;7(9):e2437.
32. Leelayoova S, Subrungruang I, Suputtamongkol Y, Worapong J, Petmitr PC, Mungthin M. Identification of genotypes of *Enterocytozoon bieneusi* from stool samples from human immunodeficiency virus-infected patients in Thailand. J Clin Microbiol. 2006;44:3001-4.
33. Karim MR, Wang R, Dong H, Zhang L, Li J, Zhang S, Rume FI, Qi M, Jian F, Sun M, Yang G, Zou F, Ning C, Xiao L. Genetic polymorphism and zoonotic potential of *Enterocytozoon bieneusi* from nonhuman primates in China. Appl Environ Microbiol. 2014;80:1893-8.
34. Ye J, Xiao L, Ma J, Guo M, Liu L, Feng Y. Anthroponotic enteric parasites in monkeys in public park, China. Emerg Infect Dis. 2012;18:1640-3.
35. Ye J, Xiao L, Li J, Huang W, Amer SE, Guo Y, Roellig D, Feng Y. Occurrence of human-pathogenic *Enterocytozoon bieneusi*, *Giardia duodenalis* and *Cryptosporidium* genotypes in laboratory macaques in Guangxi, China. Parasitol Int. 2014;63:132-7.
36. Dashti A, Santín M, Cano L, de Lucio A, Bailo B, de Mingo MH, et al. Occurrence and genetic diversity of *Enterocytozoon bieneusi* (Microsporidia) in owned and sheltered dogs and cats in Northern Spain. Parasitol Res. 2019;118:2979-2987.
37. Guo Y, Alderisio KA, Yang W, Cama V, Feng Y, Xiao L. Host specificity and source of *Enterocytozoon bieneusi* genotypes in a drinking source watershed. Appl Environ Microbiol. 2014;80:218-25.
38. da Cunha MJ, Cury MC, Santín M. Widespread presence of human-pathogenic *Enterocytozoon bieneusi* genotypes in chickens. Vet Parasitol. 2016;217:108-12.
39. Ndzi ES, Asonganyi T, Nkinin MB, Xiao L, Didier ES, Bowers LC, et al. Fast Technology Analysis Enables Identification of Species and Genotypes of Latent Microsporidia Infections in Healthy Native Cameroonians. J Eukaryot Microbiol. 2016;63:146-52.
40. Shi K, Li M, Wang X, Li J, Karim MR, Wang R, et al. Molecular survey of *Enterocytozoon bieneusi* in sheep and goats in China. Parasit Vectors. 2016;9:23.
41. Jeong DK, Won GY, Park BK, Hur J, You JY, Kang SJ, et al. Occurrence and genotypic characteristics of *Enterocytozoon bieneusi* in pigs with diarrhea. Parasitol Res. 2007;102:123-8.
42. Sarfati C, Bourgeois A, Menotti J, Liegeois F, Moyou-Somo R, Delaporte E, et al. Prevalence of intestinal parasites including microsporidia in human immunodeficiency virus-infected adults in Cameroon: a cross-sectional study. Am J Trop Med Hyg. 2006;74:162-4.
43. Agholi M, Hatam GR, Motazedian MH. HIV/AIDS-associated opportunistic protozoal diarrhea. AIDS Res Hum Retroviruses. 2013;29:35-41.
44. Ayinmode AB, Ojuromi OT, Xiao L. Molecular Identification of *Enterocytozoon bieneusi* Isolates from Nigerian Children. J Parasitol Res. 2011;2011:129542.
45. Ojuromi OT, Duan L, Izquierdo F, Fenoy SM, Oyibo WA, Del Aguila C, et al. Genotypes of *Cryptosporidium* spp. and *Enterocytozoon bieneusi* in human immunodeficiency virus-infected patients in Lagos, Nigeria. J Eukaryot Microbiol. 2016;63:414-8.
46. Karim MR, Dong H, Li T, Yu F, Li D, Zhang L, et al. Predomination and new genotypes of *Enterocytozoon bieneusi* in captive nonhuman primates in zoos in China: high genetic diversity and zoonotic significance. PLoS One. 2015;10:e0117991.
47. Sulaiman IM, Fayer R, Yang C, Santin M, Matos O, Xiao L. Molecular characterization of *Enterocytozoon bieneusi* in cattle indicates that only some isolates have zoonotic potential. Parasitol Res. 2004;92:328-34.
48. Santín M, Dargatz D, Fayer R. Prevalence and genotypes of *Enterocytozoon bieneusi* in weaned beef calves on cow-calf operations in the USA. Parasitol Res. 2012;110:2033-41.
49. Lee JH. Molecular detection of *Enterocytozoon bieneusi* and identification of a potentially human-pathogenic genotype in milk. Appl Environ Microbiol. 2008;74:1664-6.
50. Dengjel B, Zahler M, Hermanns W, Heinritzi K, Spillmann T, Thomschke A, et al. Zoonotic potential of *Enterocytozoon bieneusi*. J Clin Microbiol. 2001;39:4495-9.
51. Lobo ML, Xiao L, Cama V, Stevens T, Antunes F, Matos O. Genotypes of *Enterocytozoon bieneusi* in mammals in Portugal. J Eukaryot Microbiol. 2006;53 Suppl 1:S61-4.
52. Li W, Li Y, Song M, Lu Y, Yang J, Tao W, et al. Prevalence and genetic characteristics of *Cryptosporidium*, *Enterocytozoon bieneusi* and *Giardia duodenalis* in cats and dogs in Heilongjiang province, China. Vet Parasitol. 2015;208:125-34.
53. Li WC, Qin J, Wang K, Gu YF. Genotypes of *Enterocytozoon bieneusi* in dogs and cats in Eastern China. Iran J Parasitol. 2018;13:457-465.
54. Xu H, Jin Y, Wu W, Li P, Wang L, Li N, Feng Y, Xiao L. Genotypes of Cryptosporidium spp., *Enterocytozoon bieneusi* and *Giardia duodenalis* in dogs and cats in Shanghai, China. Parasit Vectors. 2016;9:121.
55. Abe N, Kimata I, Iseki M. Molecular evidence of *Enterocytozoon bieneusi* in Japan. J Vet Med Sci. 2009;71:217-9.
56. Pekmezci D, Pekmezci GZ, Yildirim A, Duzlu O, Inci A. Molecular Detection of Zoonotic Microsporidia in Domestic Cats in Turkey: A Preliminary Study. Acta Parasitol. 2019;64:13-18.
57. Santín M, Trout JM, Vecino JA, Dubey JP, Fayer R. Cryptosporidium, Giardia and *Enterocytozoon bieneusi* in cats from Bogota (Colombia) and genotyping of isolates. Vet Parasitol. 2006;141:334-9.
58. Karim MR, Dong H, Yu F, Jian F, Zhang L, Wang R, Zhang S, Rume FI, Ning C, Xiao L. Genetic diversity in *Enterocytozoon bieneusi* isolates from dogs and cats in China: host specificity and public health implications. J Clin Microbiol. 2014;52:3297-302.
59. Santín M, Cortés Vecino JA, Fayer R. *Enterocytozoon bieneusi* genotypes in dogs in Bogota, Colombia. Am J Trop Med Hyg. 2008;79:215-7.
60. Karim MR, Yu F, Li J, Li J, Zhang L, Wang R, Rume FI, Jian F, Zhang S, Ning C. First molecular characterization of enteric protozoa and the human pathogenic microsporidian, *Enterocytozoon bieneusi* , in captive snakes in China. Parasitol Res. 2014;113:3041-8.
61. Agholi M, Hatam GR, Motazedian MH. Microsporidia and coccidia as causes of persistence diarrhea among liver transplant children: incidence rate and species/genotypes. Pediatr Infect Dis J. 2013;32:185-7.
62. Saksirisampant W, Prownebon J, Saksirisampant P, Mungthin M, Siripatanapipong S, Leelayoova S. Intestinal parasitic infections: prevalences in HIV/AIDS patients in a Thai AIDS-care centre. Ann Trop Med Parasitol. 2009;103:573-81.
63. Prasertbun R, Mori H, Pintong AR, Sanyanusin S, Popruk S, Komalamisra C, Changbunjong T, Buddhirongawatr R, Sukthana Y, Mahittikorn A. Zoonotic potential of Enterocytozoon genotypes in humans and pigs in Thailand. Vet Parasitol. 2017;233:73-79.
64. Sokolova OI, Demyanov AV, Bowers LC, Didier ES, Yakovlev AV, Skarlato SO, Sokolova YY. Emerging microsporidian infections in Russian HIV-infected patients. J Clin Microbiol. 2011;49:2102-8.
65. Kicia M, Wesolowska M, Jakuszko K, Kopacz Z, Sak B, Květonova D, Krajewska M, Kváč M. Concurrent infection of the urinary tract with Encephalitozoon cuniculi and *Enterocytozoon bieneusi* in a renal transplant recipient. J Clin Microbiol. 2014;52:1780-2.
66. Galván AL, Sánchez AM, Valentín MA, Henriques-Gil N, Izquierdo F, Fenoy S, del Aguila C. First cases of microsporidiosis in transplant recipients in Spain and review of the literature. J Clin Microbiol. 2011;49:1301-6.
67. Li W, Deng L, Yu X, Zhong Z, Wang Q, Liu X, Niu L, Xie N, Deng J, Lei S, Wang L, Gong C, Zhou Z, Hu Y, Fu H, Xu H, Geng Y, Peng G. Multilocus genotypes and broad host-range of *Enterocytozoon bieneusi* in captive wildlife at zoological gardens in China. Parasit Vectors. 2016;9:395.
68. Chalifoux LV, Carville A, Pauley D, Thompson B, Lackner AA, Mansfield KG. *Enterocytozoon bieneusi* as a cause of proliferative serositis in simian immunodeficiency virus-infected immunodeficient macaques (*Macaca mulatta*). Arch Pathol Lab Med. 2000;124:1480-4.
69. Ye J, Xiao L, Li J, Huang W, Amer SE, Guo Y, Roellig D, Feng Y. Occurrence of human-pathogenic *Enterocytozoon bieneusi*, *Giardia duodenalis* and *Cryptosporidium* genotypes in laboratory macaques in Guangxi, China. Parasitol Int. 2014;63:132-7.
70. Abu Samra N, Thompson PN, Jori F, Zhang H, Xiao L. *Enterocytozoon bieneusi* at the wildlife/livestock interface of the Kruger National Park, South Africa. Vet Parasitol. 2012;190:587-90.
71. Zhao W, Zhang W, Yang F, Zhang L, Wang R, Cao J, Shen Y, Liu A. *Enterocytozoon bieneusi* in dairy cattle in the Northeast of China: Genetic diversity of ITS gene and evaluation of zoonotic transmission potential. J Eukaryot Microbiol. 2015;62:553-60.
72. Li J, Luo N, Wang C, Qi M, Cao J, Cui Z, Huang J, Wang R, Zhang L. Occurrence, molecular characterization and predominant genotypes of *Enterocytozoon bieneusi* in dairy cattle in Henan and Ningxia, China. Parasit Vectors. 2016;11;9:142.
73. Del Coco VF, Córdoba MA, Bilbao G, de Almeida Castro P, Basualdo JA, Santín M. First report of *Enterocytozoon bieneusi* from dairy cattle in Argentina. Vet Parasitol. 2014;199:112-5.
74. da Silva Fiuza VR, Lopes CW, de Oliveira FC, Fayer R, Santin M. New findings of *Enterocytozoon bieneusi* in beef and dairy cattle in Brazil. Vet Parasitol. 2016;216:46-51.
75. Lee JH. Prevalence and molecular characteristics of *Enterocytozoon bieneusi* in cattle in Korea. Parasitol Res. 2007;101:391-6.
76. Buckholt MA, Lee JH, Tzipori S. Prevalence of *Enterocytozoon bieneusi* in swine: an 18-month survey at a slaughterhouse in Massachusetts. Appl Environ Microbiol. 2002;68:2595-9.
77. Abe N, Kimata I. Molecular survey of *Enterocytozoon bieneusi* in a Japanese porcine population. Vector Borne Zoonotic Dis. 2010;10:425-7.
78. Li W, Diao R, Yang J, Xiao L, Lu Y, Li Y, Song M. High diversity of human-pathogenic *Enterocytozoon bieneusi* genotypes in swine in northeast China. Parasitol Res. 2014;113:1147-53.
79. Zhao W, Zhang W, Yang F, Cao J, Liu H, Yang D, Shen Y, Liu A. High prevalence of *Enterocytozoon bieneusi* in asymptomatic pigs and assessment of zoonotic risk at the genotype level. Appl Environ Microbiol. 2014;80:3699-3707.
80. Sak B, Kvác M, Hanzlíková D, Cama V. First report of *Enterocytozoon bieneusi* infection on a pig farm in the Czech Republic. Vet Parasitol. 2008;153:220-4.
81. Wagnerová P, Sak B, Květoňová D, Buňatová Z, Civišová H, Maršálek M, Kváč M. *Enterocytozoon bieneusi* and *Encephalitozoon cuniculi* in horses kept under different management systems in the Czech Republic. Vet Parasitol. 2012 21;190:573-7.
82. Laatamna AE, Wagnerová P, Sak B, Květoňová D, Xiao L, Rost M, McEvoy J, Saadi AR, Aissi M, Kváč M. Microsporidia and *Cryptosporidium* in horses and donkeys in Algeria: detection of a novel *Cryptosporidium hominis* subtype family (Ik) in a horse. Vet Parasitol. 2015;208:135-42.
83. Qi M, Wang R, Wang H, Jian F, Li J, Zhao J, Dong H, Zhu H, Ning C, Zhang L. *Enterocytozoon bieneusi* genotypes in grazing horses in china and their zoonotic transmission potential. J Eukaryot Microbiol. 2016;63:591-7.
84. Santín M, Vecino JA, Fayer R. A zoonotic genotype of *Enterocytozoon bieneusi* in horses. J Parasitol. 2010;96:157-61.
85. Mori H, Mahittikorn A, Thammasonthijarern N, Chaisiri K, Rojekittikhun W, Sukthana Y. Presence of zoonotic *Enterocytozoon bieneusi* in cats in a temple in central Thailand. Vet Parasitol. 2013;197:696-701.
86. Prado JBF, Ramos CADN, Fiuza VRDS, Terra VJB. Occurrence of zoonotic *Enterocytozoon bieneusi* in cats in Brazil. Rev Bras Parasitol Vet. 2019;28:80-90.
87. Piekarska J, Kicia M, Wesołowska M, Kopacz Ż, Gorczykowski M, Szczepankiewicz B, Kváč M, Sak B. Zoonotic microsporidia in dogs and cats in Poland. Vet Parasitol. 2017;246:108-111.
88. Němejc K, Sak B, Květoňová D, Hanzal V, Janiszewski P, Forejtek P, Rajský D, Kotková M, Ravaszová P, McEvoy J, Kváč M. Prevalence and diversity of *Encephalitozoon* spp. and *Enterocytozoon bieneusi* in wild boars (*Sus scrofa*) in Central Europe. Parasitol Res. 2014;113:761-7.
89. Zhao GH, Du SZ, Wang HB, Hu XF, Deng MJ, Yu SK, Zhang LX, Zhu XQ. First report of zoonotic *Cryptosporidium* spp., *Giardia intestinalis* and *Enterocytozoon bieneusi* in golden takins (*Budorcas taxicolor bedfordi*). Infect Genet Evol. 2015;34:394-401.
90. Sulaiman IM, Fayer R, Lal AA, Trout JM, Schaefer FW 3rd, Xiao L. Molecular characterization of microsporidia indicates that wild mammals Harbor host-adapted *Enterocytozoon* spp. as well as human-pathogenic *Enterocytozoon bieneusi* . Appl Environ Microbiol. 2003;69:4495-501.
91. Yang Y, Lin Y, Li Q, Zhang S, Tao W, Wan Q, Jiang Y, Li W. Widespread presence of human-pathogenic *Enterocytozoon bieneusi* genotype D in farmed foxes (*Vulpes vulpes*) and raccoon dogs (*Nyctereutes procyonoides*) in China: first identification and zoonotic concern. Parasitol Res. 2015;114:4341-8.
92. Zhao W, Zhang W, Yang Z, Liu A, Zhang L, Yang F, Wang R, Ling H. Genotyping of *Enterocytozoon bieneusi* in farmed blue foxes (*Alopex lagopus*) and raccoon dogs (*Nyctereutes procyonoides*) in China. PLoS One. 2015;10:e0142611.
93. Sak B, Kváč M, Květoňová D, Albrecht T, Piálek J. The first report on natural *Enterocytozoon bieneusi* and Encephalitozoon spp. infections in wild East-European House Mice (*Mus musculus musculus*) and West-European House Mice (*M. m. domesticus*) in a hybrid zone across the Czech Republic-Germany border. Vet Parasitol. 2011;178:246-50.
94. Müller MG, Kinne J, Schuster RK, Walochnik J. Outbreak of microsporidiosis caused by *Enterocytozoon bieneusi* in falcons. Vet Parasitol. 2008;152:67-78.
95. Pirestani M, Sadraei J, Forouzandeh M. Molecular characterization and genotyping of human related microsporidia in free-ranging and captive pigeons of Tehran, Iran. Infect Genet Evol. 2013;20:495-9.
96. Sak B, Brady D, Pelikánová M, Květoňová D, Rost M, Kostka M, Tolarová V, Hůzová Z, Kváč M. Unapparent microsporidial infection among immunocompetent humans in the Czech Republic. J Clin Microbiol. 2011;49:1064-70.
97. Zhang X, Wang Z, Su Y, Liang X, Sun X, Peng S, Lu H, Jiang N, Yin J, Xiang M, Chen Q. Identification and genotyping of *Enterocytozoon bieneusi* in China. J Clin Microbiol. 2011;49:2006-8.
98. Fayer R, Santín M, Trout JM. *Enterocytozoon bieneusi* in mature dairy cattle on farms in the eastern United States. Parasitol Res. 2007;102:15-20.
99. Santín M, Trout JM, Fayer R. *Enterocytozoon bieneusi* genotypes in dairy cattle in the eastern United States. Parasitol Res. 2005;97:535-8.
100. Santín M, Fayer R. A longitudinal study of *Enterocytozoon bieneusi* in dairy cattle. Parasitol Res. 2009;105:141-4.
101. Ma J, Cai J, Ma J, Feng Y, Xiao L. *Enterocytozoon bieneusi* genotypes in yaks (*Bos grunniens*) and their public health potential. J Eukaryot Microbiol. 2015 Jan-Feb;62(1):21-5.
102. Jiang Y, Tao W, Wan Q, Li Q, Yang Y, Lin Y, et al. Zoonotic and potentially host-adapted *Enterocytozoon bieneusi* genotypes in sheep and cattle in northeast china and an increasing concern about the zoonotic importance of previously considered ruminant-adapted genotypes. Appl Environ Microbiol. 2015;81:3326-35.
103. Zhao W, Zhang W, Yang D, Zhang L, Wang R, Liu A. Prevalence of *Enterocytozoon bieneusi* and genetic diversity of ITS genotypes in sheep and goats in China. Infect Genet Evol. 2015;32:265-70.
104. Rinder H, Thomschke A, Dengjel B, Gothe R, Löscher T, Zahler M. Close genotypic relationship between *Enterocytozoon bieneusi* from humans and pigs and first detection in cattle. J Parasitol. 2000;86:185-8.
105. Reetz J, Nöckler K, Reckinger S, Vargas MM, Weiske W, Broglia A. Identification of *Encephalitozoon cuniculi* genotype III and two novel genotypes of *Enterocytozoon bieneusi* in swine. Parasitol Int. 2009;58:285-92.
106. Fiuza VR, Oliveira FC, Fayer R, Santín M. First report of *Enterocytozoon bieneusi* in pigs in Brazil. Parasitol Int. 2015;64:18-23.
107. Li W, Tao W, Jiang Y, Diao R, Yang J, Xiao L. Genotypic distribution and phylogenetic characterization of *Enterocytozoon bieneusi* in diarrheic chickens and pigs in multiple cities, China: potential zoonotic transmission. PLoS One. 2014;9:e108279.
108. Wan Q, Lin Y, Mao Y, Yang Y, Li Q, Zhang S, et al. High prevalence and widespread distribution of zoonotic *Enterocytozoon bieneusi* genotypes in swine in Northeast China: implications for public health. J Eukaryot Microbiol. 2016;63:162-70.
109. Breitenmoser AC, Mathis A, Bürgi E, Weber R, Deplazes P. High prevalence of *Enterocytozoon bieneusi* in swine with four genotypes that differ from those identified in humans. Parasitology. 1999;118:447-53.
110. Lallo MA, Calábria P, Milanelo L. *Encephalitozoon* and *Enterocytozoon* (Microsporidia) spores in stool from pigeons and exotic birds: microsporidia spores in birds. Vet Parasitol. 2012;190:418-22.
111. Juránková J, Kamler M, Kovařčík K, Koudela B. *Enterocytozoon bieneusi* in Bovine Viral Diarrhea Virus (BVDV) infected and noninfected cattle herds. Res Vet Sci. 2013;94(1):100-4.
112. Fayer R, Santin M, Macarisin D. Detection of concurrent infection of dairy cattle with *Blastocystis*, *Cryptosporidium*, *Giardia*, and *Enterocytozoon* by molecular and microscopic methods. Parasitol Res. 2012;111:1349-55.
113. Santin M, Fayer R. *Enterocytozoon bieneusi*, *Giardia*, and *Cryptosporidium* infecting white-tailed deer. J Eukaryot Microbiol. 2015;62:34-43.
114. Widmer G, Dilo J, Tumwine JK, Tzipori S, Akiyoshi DE. Frequent occurrence of mixed *Enterocytozoon bieneusi* infections in humans. Appl Environ Microbiol. 2013;79:5357-62.
115. Santín M, Calero-Bernal R, Carmena D, Mateo M, Balseiro A, Barral M, et al. Molecular characterization of *Enterocytozoon bieneusi* in wild carnivores in Spain. J Eukaryot Microbiol. 2018;65:468-474.
116. Baroudi D, Zhang H, Amer S, Khelef D, Roellig DM, Wang Y, et al. Divergent *Cryptosporidium parvum* subtype and *Enterocytozoon bieneusi* genotypes in dromedary camels in Algeria. Parasitol Res. 2018;117:905-910.
