# Supplementary material for: First identification of genotypes of Enterocytozoon bieneusi (Microsporidia) among symptomatic and asymptomatic children in Mozambique
Source: PLoS Negl Trop Dis. 2020 Jun 30;14(6):e0008419. doi: 10.1371/journal.pntd.0008419 (PMC7357779; doi:10.1371/journal.pntd.0008419)
Supplement: Attachment — Submitted filename: RewierComments_PNTD-D-20-00113.docx [file pntd.0008419.s007.docx]

**Manuscript Number: PNTD-D-20-00113**

Title: First identification of genotypes of *Enterocytozoon bieneusi* (Microsporidia) among symptomatic and asymptomatic children in Mozambique

Authors: Aly Salimo Muadica, Augusto E. Messa Jr., Alejandro Dashti, Mónica Santin, Filomena Manjate, Percina Chirinda, Marcelino Garrine4, Delfino Vubil, Sozinho Acácio, Pamela C. Köster, Begoña Bailo, Tacilta Nhampossa, Rafael Calero, Berna, Jason Mwenda, Inácio Mandomando, David Carmena

Article Type: Research Article

**Comments and suggestions:**

Overall the manuscript by Muadica *et al*. provides useful information on *Enterocytozoon bieneusi* genotyping in children from Mozambique. Although the manuscript is not of sufficient originality, and quality I think the paper might be of interest to the *PLOS Neglected Tropical Diseases* journal's readers with major revisions.

The authors may wish to consider the following comments and suggestions:

**1.** In my opinion, **the manuscript should be shortened**. Some suggestions that would improve the reading of the manuscript:

1.1 The Methods section should be quite shortened.

1.2 Also, the Results section can be shortened. For instance, the authors can remove extra text from of the manuscript (e.g. lines 362 to 381) whenever the data cited are included in tables 2 and/or 3 in order to not duplicate information and to make the manuscript more informative and accurate.

1.3. In my opinion, Figure 1 do not have relevant information and could be removed from the manuscript or add to supporting information section

**2.** Why the authors did not consider performing a statistical analysis of all the epidemiological, clinical and laboratory data obtained for the 1,247 children enrolled in this study?

The application of statistical analysis (for instance multivariate analysis) can contribute to clarify and explain relationships between different variables that could be associated with data obtained and **is mandatory for quality improvement of the study**.

3. The authors cited in the text of the manuscript (page 31, lines 421-424): “*Overall our prevalence is similar to those previously documented in immunocompetent children populations (range: 0‒9%) in other African countries including the Democratic Republic of São Tomé and Principe [27], Mali [28], Nigeria [29], South Africa [30], and Zambia [31]…*”

However, these citation is not correct as the value of 0,7% of *Enterocytozoon bieneusi* infection obtained in this study is not similar but lower than the values obtained in the referred studies, namely 5,2% and 8,9% in Democratic Republic of São Tomé and Principe [27], 13,1% in Mali [28], 9,3% in Nigeria [29] and 4,5- 12,9% in South Africa [30], **The authors should check carefully and correct the issues cited in the manuscript.**

4. The authors did not discuss any of the epidemiological, clinical variables collected that could be potentially associated with the laboratory data obtained in this study. It was important to **evaluate** if there was **any risk factor among the variables** studied **associated** with the **microsporidia infection** in this population.
