# Supplementary material for: First identification of genotypes of Enterocytozoon bieneusi (Microsporidia) among symptomatic and asymptomatic children in Mozambique
Source: PLoS Negl Trop Dis. 2020 Jun 30;14(6):e0008419. doi: 10.1371/journal.pntd.0008419 (PMC7357779; doi:10.1371/journal.pntd.0008419)
Supplement: Attachment — Submitted filename: Letter to reviewers PNTD-D-20-00113.docx [file pntd.0008419.s008.docx]

PNTD-D-20-00113

**First identification of genotypes of *Enterocytozoon bieneusi* (Microsporidia) among symptomatic and asymptomatic children in Mozambique**

Muadica, Messa Jr., et al.

Dear Dr. Le and Dr. Reynolds,

Thank you for your favourable preliminary appraisal on Ms. PNTD-D-20-00113, and for making available to us the Editor´s and Reviewers' comments, which we have considered carefully. Please see below a thorough point-by-point response to each issue raised by the Editor and Reviewers. Changes introduced in the main body of the text have been highlighted in red for better identification.

**Editor´s comments and suggestions**

Overall, the manuscript by Muadica et al. provides useful information on *Enterocytozoon bieneusi* genotyping in children from Mozambique. Although the manuscript is not of sufficient originality, and quality I think the paper might be of interest to the PLOS Neglected Tropical Diseases journal's readers with major revisions. The authors may wish to consider the following comments and suggestions:

1. In my opinion, the manuscript should be shortened. Some suggestions that would improve the reading of the manuscript:

The Methods section should be quite shortened. Also, the Results section can be shortened. For instance, the authors can remove extra text from of the manuscript (e.g. lines 362 to 381) whenever the data cited are included in tables 2 and/or 3 in order to not duplicate information and to make the manuscript more informative and accurate.

Response: Following the Editor´s recommendation, the overall length of the manuscript has been reduced. Particularly, the Methods section has been significantly shortened in subsections covering Ethics statement, Study design, Collection of stool samples in Zambézia and Manhiça, and DNA extraction. In Results, redundant information has been removed avoiding repeating information already provided in Tables and supplementary material.

2. In my opinion, Figure 1 do not have relevant information and could be removed from the manuscript or add to supporting information section

Response: The Figure 1 has been presented as supplementary data (S1 Fig) in the revised version.

3. Why the authors did not consider performing a statistical analysis of all the epidemiological, clinical and laboratory data obtained for the 1,247 children enrolled in this study? The application of statistical analysis (for instance multivariate analysis) can contribute to clarify and explain relationships between different variables that could be associated with data obtained and is mandatory for quality improvement of the study.

Response: We agree with the Editor comment and to sort this issue out, we contacted and invited Dr Sooria Balasegaram (Public Health England, UK) to participate in the project assisting with statistical analyses. A statistical analysis section is now included in Methods and results derived from it are summarized in a new Table (Table 4). This Information is appropriately commented and discussed in the Abstract, Results, and Discussion sections. A new reference (#25) has been added to the reference list to back up the methodology used. Dr. Balasegaram has been added as co-author and her contribution fully acknowledged in the Author Contribution section.

4. The authors cited in the text of the manuscript (page 31, lines 421-424): “Overall our prevalence is similar to those previously documented in immunocompetent children populations (range: 0‒9%) in other African countries including the Democratic Republic of São Tomé and Principe [27], Mali [28], Nigeria [29], South Africa [30], and Zambia [31]…” However, these citation is not correct as the value of 0,7% of *Enterocytozoon bieneusi* infection obtained in this study is not similar but lower than the values obtained in the referred studies, namely 5,2% and 8,9% in Democratic Republic of São Tomé and Principe [27], 13,1% in Mali [28], 9,3% in Nigeria [29] and 4,5-12,9% in South Africa [30], The authors should check carefully and correct the issues cited in the manuscript.

Response: We thank the editor for this observation, and we apologize for this inaccuracy in the text. We have now addressed this issue in the discussion section.

5. The authors did not discuss any of the epidemiological, clinical variables collected that could be potentially associated with the laboratory data obtained in this study. It was important to evaluate if there was any risk factor among the variables studied associated with the microsporidia infection in this population.

Response: As suggested this has been done and added to the paper. See our answer to Comment #3.

**Reviewer's Responses to Questions**

**Methods**

**Reviewer #1**

The manuscript by Salimo Muadica et al report on an extensive survey of *Enterocytozoon bieneusi* infection in children in Mozambique. These studies are difficult to conduct and the authors deserve credit for surveying a relatively large number of children.

Response: We thank Reviewer #1 for his preliminary positive appraisal.

**Reviewer #2**: (No Response)

**Reviewer #3**:

In the epigraphs “Collection of stool samples in Zambézia province” and “Collection of stool samples in the Manhiça district, Maputo province”, in my opinion is confused the treatment and sample processing (lines 188-190 and lines 229-231, respectively). The authors could clarify or homogenize this step.

Response: The authors would like to clarify that data presented in this manuscript come from two independent studies conducted by different research teams and following different sampling designs. This is the reason why the description of sampling sites and sampling procedures, including sample processing differ between Zambézia and Manhiça. This issue has been now clarified at the end of the Study design subsection. We decided to jointly present the obtained results from these surveys in order to i) provide more representative data at the national level, and ii) increase sample size to improve statistical power.

Line 243: “DNA extraction”. Add: “and purification”.

Response: Corrected as per requested.

**Results**

Reviewer #1

Perhaps the most important contribution of the study is the apparent absence of association between diarrhea and Eb.

Response: We agree with Reviewer #1. We have now highlighted this point in the conclusion section.

**Reviewer #2**: (No Response)

**Reviewer #3**:

Line 328: “Overall, *E. bieneusi* was detected by PCR”. Add: nested.

Response: Corrected as per requested.

Table 1: Include in the table foot (legend), TEM (transmission electron microscopy).

Response: The legend was added as per requested. We have also tidy up the whole Table to improve clarity and added minor modifications.

Table 3: Remove in table foot “NA: not applicable”, I think it is not used.

Response: The term “NA” has been removed from Table 3.

S3 Table: Remove in table foot “NA: not applicable”, I think it is not used.

Response: The term “NA” has been removed from S3 Table.

**Conclusions**

**Reviewer #1**

Given the size of the study, the small number of positives is worth reporting, but statements comparing the prevalence in the 2 study sites (line 328, 412) and between this and previously published reports (lines 414-425) may not be warranted unless supported by a statistical analysis.

Response: please see our detailed answer to Comment #3 by the Editor. We have included confidence intervals around our estimate, but with only 9 positive samples, but agree that we should be circumspect in making comparisons.

Similarly, the statement on line 333 about the absence of mixed infections seems problematic given the PCR and sequencing method used in this study; see for instance Widmer G, Dilo J, Tumwine JK, Tzipori S, Akiyoshi DE. Appl Environ Microbiol. 2013, Sep;79(17):5357-62.

Response: We agree with reviewer that PCR Sanger sequencing is not the best method to detect mixed infection. We have acknowledged the issue of mixed infections at the beginning of the last paragraph of the Discussion section and reference Widmer et al., Appl Environ Microbiol. 2013;79:5357-5362 has added to the reference list.

**Reviewer #2**: (No Response)

**Reviewer #3:**

Lines 455-461: Comment: The authors should cite or comment the importance of the water transmission of the parasite for a better knowledge of the transmission pathways in the context in which the study was carried out.

Response: Following Reviewer # advice, this issue has been addressed in the last paragraph of the discussion section by adding the sentence “Environmental (water) samples should be also considered as a source of infection by microsporidia, as *E. bieneusi* has been demonstrated to be involved in waterborne outbreaks of gastrointestinal disease [51].

A new reference (#51) has been added to back up this statement.

**Editorial and Data Presentation Modifications**

**Reviewer #1**

The manuscript is generally well written, but some additional editing seems warranted. See following examples:

Line 206-219. Consider reporting enrolment criteria as a numbered or bulleted list. Text using numerous parentheses and nested parentheses is difficult to follow.

Response: Following Reviewer 1 advice, the text has been now shortened and rephrased to improve clarity and fluidity. See also our answer to Comment #1 by the Editor.

Line 103. …capable of colonizing/infecting a broad spectrum…

Response: Corrected.

Line 110. Nearly 500 genotypes

Response: Corrected.

Line 113. …mammalian species, including humans…

Response: Corrected.

Line 126. …could be more frequent that anticipated. Despite the progress…

Response: Corrected.

Line 156. Delete “the” (…under reference number…)

Response: Corrected.

Line 186. …seeking medical attention… (no “for” preposition)

Response: Corrected.

Line 192. …children were between 3 and 14 years old.

Response: Corrected.

Line 201. Delete “continuous” (it’s implied in the sentence); something is missing after (HDDS), like program or survey?

Response: This paragraph has been deleted from the text to comply with the Editor´s request to shorten the length of this section of the manuscript.

Line 202. Start new sentence: This survey covers approximately…

Response: This paragraph has been deleted from the text to comply with the Editor´s request to shorten the length of this section of the manuscript.

Line 208. Peripheral health facilities

Response: Corrected.

Line 224. …were enrolled starting in April 2017.

Response: Corrected.

Line 230. Testing for enteric pathogens

Response: This paragraph has been deleted from the text to comply with the Editor´s request to shorten the length of this section of the manuscript.

Line 249. In Manhica, DNA was manually extracted and purified using the QIA….

etc.

Response: Corrected

**Reviewer #2**: (No Response)

**Reviewer #3**

Keywords: Add: novel genotype.

Response: The term “genotype” has been added to the list of keywords.

Line 33: “*Enterocytozoon bieneusi* (Phylum Microsporidia)”. Comment: With the new classification of the Eukaryotes, in my opinion the Microsporidia (Fungi) is not a Phylum.

Reference: 1: Adl SM, Bass D, Lane CE, Lukeš J, Schoch CL, Smirnov A, Agatha S, Berney C,Brown MW, Burki F, Cárdenas P, Čepička I, Chistyakova L, Del Campo J, Dunthorn M,Edvardsen B, Eglit Y, Guillou L, Hampl V, Heiss AA, Hoppenrath M, James TY, Karnkowska A, Karpov S, Kim E, Kolisko M, Kudryavtsev A, Lahr DJG, Lara E, Le Gall L, Lynn DH, Mann DG, Massana R, Mitchell EAD, Morrow C, Park JS, Pawlowski JW, Powell MJ, Richter DJ, Rueckert S, Shadwick L, Shimano S, Spiegel FW, Torruella G, Youssef N, Zlatogursky V, Zhang Q. Revisions to the Classification, Nomenclature, and Diversity of Eukaryotes. J Eukaryot Microbiol. 2019 Jan; 66(1):4-119.

Response: As suggested, to avoid controversy and since this is not the topic of this study the term “Phylum Microsporidia” was deleted from the text.

Line 92: “Microsporidia comprises 200 genera and nearly 1,500 species of spore-forming parasites”. Add: “Microsporidia (Fungi) comprises 200 genera and nearly 1,500 species of spore-forming parasites”.

Response: Corrected.

- Lines 95-96: “causing gastrointestinal infections globally”. Add: “and other disorders and pathologies such as in lung”.

Reference: “del Águila C, Lopez-Velez R, Fenoy S, Turrientes C, Cobo J, Navajas R, Visvesvara GS, Croppo GP, Da Silva AJ, Pieniazek NJ. Identification of *Enterocytozoon bieneusi* spores in respiratory samples from an AIDS patient with a 2-year history of intestinal microsporidiosis. J Clin Microbiol. 1997, Jul; 35(7): 1862-6”.

Response: Following Reviewer #3 advice, this sentence has been rephrased as “Extraintestinal disorders and pathologies have also been reported, mainly in lung”. The reference del Águila et al. 1997 has been added to the reference list to back up this statement.

Lines 105-107: “Infections are acquired through ingestion of food or water contaminated with *E. bieneusi* spores, or through direct contact with faeces of infected persons and animals”. Add: soils.

Response: The expression “with contaminated soils” have been now added.

Lines 118-120: “GEMS included in their study enteric protozoan parasites *Giardia duodenalis*, *Cryptosporidium* spp., and *Entamoeba histolytica*, but not *E. bieneusi*”. Comment: *E. bieneusi* is not a protozoa parasite. Add: enteric parasites, for example.

Response: To avoid confusion the expression ´…but not *E. bieneusi*´ has been now replaced by the expression ´…but not the microsporidia *E. bieneusi*´.

Line 130: “asymptomatic paediatric populations”. Change for “pediatric”.

Response: Please note that the whole manuscript is written in British English, in which the correct term is “paediatric”. The term “pediatric” is American English.

Materials and Methods. In the epigraphs “Collection of stool samples in Zambézia province” and “Collection of stool samples in the Manhiça district, Maputo province”, in my opinion is confused the treatment and sample processing (lines 188-190 and lines 229-231, respectively). The authors could clarify or homogenize this step.

Response: Data presented in this manuscript come from two independent studies conducted by different research teams and following different sampling designs. This is the reason why the description of sampling sites and sampling procedures, including sample processing differ between Zambézia and Manhiça. This issue has been now clarified at the end of the study design section. We decided to jointly present the obtained results from these surveys in order to i) provide more representative data at the national level, and ii) increase sample size to improve statistical power.

Line 243: “DNA extraction”. Add: “and purification”.

Response: Corrected.

Line 328: “Overall, *E. bieneusi* was detected by PCR”. Add: nested.

Response: Corrected as per requested by Reviewer #3.

Lines 455-461: Comment: The authors should cite or comment the importance of the water transmission of the parasite for a better knowledge of the transmission pathways in the context in which the study was carried out.

Response: Following Reviewer´s #3 advice, this issue has been addressed in the discussion section by adding the sentence “Environmental (water) samples should be also considered as a source of infection by microsporidia, as *E. bieneusi* has been demonstrated to be involved in waterborne outbreaks of gastrointestinal disease [51]. A new reference (#51) has been added to back up this statement.

Table 1: Include in the table foot (legend), TEM (transmission electron microscopy).

Response: Added as per requested by Reviewer #3.

Table 3: Remove in table foot “NA: not applicable”, I think it is not used.

Response: The term “NA” has been removed from Table 3.

S3 Table: Remove in table foot “NA: not applicable”, I think it is not used.

Response: The term “NA” has been removed from S3 Table.

References:

Add: “Adl SM, Bass D, Lane CE, Lukeš J, Schoch CL, Smirnov A, Agatha S, Berney C,Brown MW, Burki F, Cárdenas P, Čepička I, Chistyakova L, Del Campo J, Dunthorn M,Edvardsen B, Eglit Y, Guillou L, Hampl V, Heiss AA, Hoppenrath M, James TY, Karnkowska A, Karpov S, Kim E, Kolisko M, Kudryavtsev A, Lahr DJG, Lara E, Le Gall L, Lynn DH, Mann DG, Massana R, Mitchell EAD, Morrow C, Park JS, Pawlowski JW, Powell MJ, Richter DJ, Rueckert S, Shadwick L, Shimano S, Spiegel FW, Torruella G, Youssef N, Zlatogursky V, Zhang Q. Revisions to the Classification, Nomenclature, and Diversity of Eukaryotes. J Eukaryot Microbiol. 2019 Jan; 66(1):4-119”.

Response: As suggested, to avoid controversy and since this is not the topic of this study the term “Phylum Microsporidia” was deleted from the text.

- Add: “del Águila C, Lopez-Velez R, Fenoy S, Turrientes C, Cobo J, Navajas R, Visvesvara GS, Croppo GP, Da Silva AJ, Pieniazek NJ. Identification of Enterocytozoon bieneusi spores in respiratory samples from an AIDS patient with a 2-year history of intestinal microsporidiosis. J Clin Microbiol. 1997, Jul; 35(7): 1862-6”.

Response: The reference del Águila et al. 1997 has been added to the reference list.

**Summary and General Comments**

*Reviewer #1*

The apparent absence of association between diarrhea and Eb is worth reporting. Some of the conclusions should be supported by statistical tests.

This has now been done.

**Reviewer #2**: (No Response)

**Reviewer #3**

The main objective of the manuscript is very interesting with promising results. In my opinion would have been interesting to investigate the presence of other species of Microsporidia (for example *Encephalitozoon* spp.) using others complementary techniques such as staining methods or immunological techniques. Obviously, the authors want to study only the genotypes of *E. bieneusi* in children in Africa (Mozambique) as main objective. But these additional techniques (staining methods or immunological techniques) could contribute to knowledge of the prevalence of other species of Microsporidia and provide us of a valuable information for this study or for new studies in the future.

Reviewer #3 is right. We initially focused on *E. bieneusi* because it is a well-known opportunistic pathogen in immunocompromised patients, and we wanted to investigate its presence in asymptomatic individuals. However, recent molecular epidemiological studies are revealing that *Encepahlocytozoon cuniculi*, E. *intestinalis* and *E. hellem* are apparently more prevalent than *E. bieneusi* in individuals without clinical manifestations (see for instance Greigert et al. PLoS One. 2018;13(10):e0204576; Aissa et al. Médecine et Santé Tropicales. 2017;27(3):281-285 and Sak et al. J Clin Microbiol. 2011;49(3):1064‐1070). This is something of which we were not aware at the moment of designing and conducting the present study. Although the literature is still scarce, it seems that subclinical infections by the above-mentioned microsporidia species are more frequent than anticipated. Taking advantage of the large panel of DNA samples that we have collected, rest assured that we will re-test them for the presence of *E. cuniculi*, *E. intestinalis* and *E. hellem*.

I enjoyed reviewing this article. It is extensive with detailed information with a complete review of the prevalence of *E. bieneusi* and its genotypes in Africa. The results are interesting highlighting the description of a novel genotype. I encourage the authors to continue investigating the circulation and prevalence of this novel genotype and those already described in Africa.

We thank Reviewer #3 for his/her encouragement and useful criticisms and suggestions.
